# Exosome‐Based Mitochondrial Delivery of circRNA mSCAR Alleviates Sepsis by Orchestrating Macrophage Activation

**DOI:** 10.1002/advs.202205692

**Published:** 2023-03-25

**Authors:** Li Fan, Li Yao, Zhelong Li, Zhuo Wan, Wenqi Sun, Shuo Qiu, Wei Zhang, Dan Xiao, Liqiang Song, Guodong Yang, Yi Zhang, Mengying Wei, Xuekang Yang

**Affiliations:** ^1^ Department of Burns and Cutaneous Surgery Xijing Hospital Fourth Military Medical University Xi'an 710032 China; ^2^ Department of Pathology Xi'an No. 3 Hospital The Affiliated Hospital of Northwest University Xi'an 710018 China; ^3^ Department of Ultrasound Diagnostics Tangdu Hospital, Fourth Military Medical University Xi'an 710038 China; ^4^ Department of Hematology Tangdu Hospital Fourth Military Medical University Xi'an 710038 China; ^5^ Department of Respiratory Medicine Tangdu Hospital Fourth Military Medical University Xi'an 710038 China; ^6^ Department of Pulmonary and Critical Care Medicine Xijing Hospital, Fourth Military Medical University Xi'an 710032 China; ^7^ The State Laboratory of Cancer Biology Department of Biochemistry and Molecular Biology Fourth Military Medical University Xi'an 710032 China; ^8^ Department of Dental Clinical Diagnostics School of Stomatology Fourth Military Medical University Xi'an 710032 China

**Keywords:** circRNA mSCAR, exosome, macrophage polarization, mitochondrial targeted delivery, sepsis

## Abstract

Sepsis is one of the most common causes of death, which is closely related to the uncontrolled systemic inflammation. Dysregulation of M1 macrophage polarization is the primary contributor to serious inflammation. In this study, it is revealed that the murine homologue of circRNA SCAR (steatohepatitis‐associated circRNA ATP5B regulator), denoted as circRNA mSCAR hereafter, decreases in the macrophages of septic mice, which correlates with the excessive M1 polarization. To restore circRNA mSCAR in mitochondria, exosomes encapsulated with circRNA mSCAR are further electroporated with poly‐D‐lysine‐graft‐triphenylphosphine (TPP‐PDL), and thus TPP‐PDL facilitates the bound circRNA delivered into mitochondria when the exosomes engulf by the recipient cells. In in vivo septic mouse model and in vitro cell model, it is shown that the exosome‐based mitochondria delivery system delivers circRNA mSCAR into mitochondria preferentially in the macrophages, favoring macrophage polarization toward M2 subtype. Accordingly, the systemic inflammation is attenuated by exosome‐based mitochondrial delivery of circRNA mSCAR, together with alleviated mortality. Collectively, the results uncover the critical role of circRNA mSCAR in sepsis, and provide a promising approach to attenuate sepsis via exosome‐based mitochondrial delivery of circRNA mSCAR.

## Graphical abstract

Exosomes encapsulated with circRNA mSCAR, a mitochondrial circRNA promoting M2 polarization via decreasing mitochondrial reactive oxygen species (mtROS), are additionally loaded with TPP‐PDL. Following in vivo delivery, circRNA mSCARs in the exosomes are preferentially delivered into the mitochondria of macrophage, promoting M2 macrophage polarization and thus attenuating sepsis.

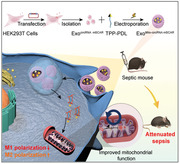

## 1 Introduction

Sepsis is a highly complex and lethal syndrome, affecting millions of people worldwide each year.^[^ ^1^, ^2^ ^]^ A large series of experiments have established that patients with severe infection tend to have a hyperinflammatory response, which is the key contributor of multi‐organ dysfunction.^[^ ^3^, ^4^, ^5^ ^]^ Anti‐inflammatory interventions have been regarded as the effective methods to treat sepsis, and substantial strategies to alleviate inflammation have been explored.^[^ ^6^, ^7^, ^8^, ^9^, ^10^ ^]^ However, the results of blocking inflammatory cascade in sepsis is controversial. Thus, understanding the mechanism of inflammation and developing new treatments are necessary to improve the prognosis of sepsis.

Systemic inflammatory response syndrome of sepsis is associated with the activation of immune cells such as neutrophils, macrophages, and nature killer cells.^[^ ^11^ ^]^ Among them, macrophages are the most abundant immune cells in many tissues and one of the first responders to damage.^[^ ^12^ ^]^ When faced with high bacterial load, proinflammatory macrophages are overactivated, contributing to the progression of sepsis.^[^ ^13^ ^]^ Besides, insufficient number of anti‐inflammatory macrophages, which has been shown to exhibit critical regulatory activity at all stages of repair,^[^ ^14^ ^]^ is another factor leading to the development of sepsis. Thus, regulating macrophage polarization is likely to be a potential therapeutic strategy for sepsis.

Studies have shown that mitochondria function importantly in macrophage polarization.^[^ ^15^, ^16^, ^17^ ^]^ Recent years, there has been growing evidence showing that mitochondrial DNA (mtDNA) can encode non‐coding RNAs with potent regulatory functions, such as long non‐coding RNAs, miRNAs, and circular RNAs (circRNAs).^[^ ^18^, ^19^, ^20^ ^]^ Among them, circRNAs are most stable for they have neither 5’ to 3’ polarity nor a polyadenylated tail.^[^ ^21^, ^22^ ^]^ Thus, therapeutic delivery of mitochondrial circRNA would be promising in tuning mitochondrial function, though challenged due to the double‐layered membrane structure of mitochondria.^[^ ^23^ ^]^

**Scheme 1:**
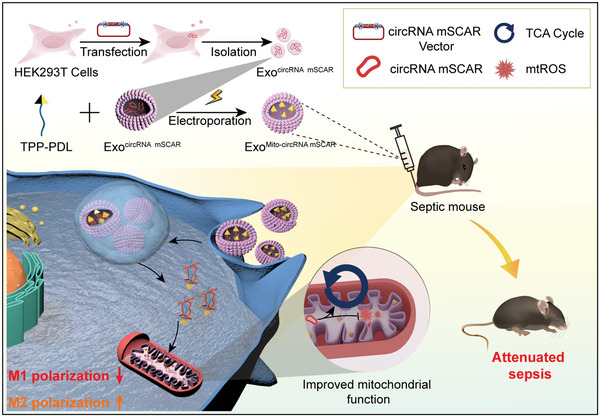
Exosomes encapsulated with circRNA mSCAR, a mitochondrial circRNA promoting M2 polarization via decreasing mtROS, are additionally loaded with TPP‐PDL. Following in vivo delivery, circRNA mSCARs in the exosomes are preferentially delivered into the mitochondria of macrophage, promoting M2 macrophage polarization and thus attenuating sepsis.

Herein, we revealed that reduction of circRNA mSCAR in macrophages of septic mice is closely related to M1 macrophage polarization. Encapsulation of circRNA mSCAR and TPP‐PDL together into exosomes could deliver circRNA mSCAR into mitochondria of macrophages, and thus increases polarization of M2 and ameliorates sepsis‐induced organ injury (**Scheme** **1**). Our study reveals a mechanism by which circRNA mSCAR can orchestrate macrophage polarization and highlights the therapeutic potential of mitochondria targeted delivery system for sepsis and other inflammatory diseases.

## 2 Results and Discussion

### 2.1 Overactivated M1 Polarization in Murine Sepsis Model

**Figure 1:**
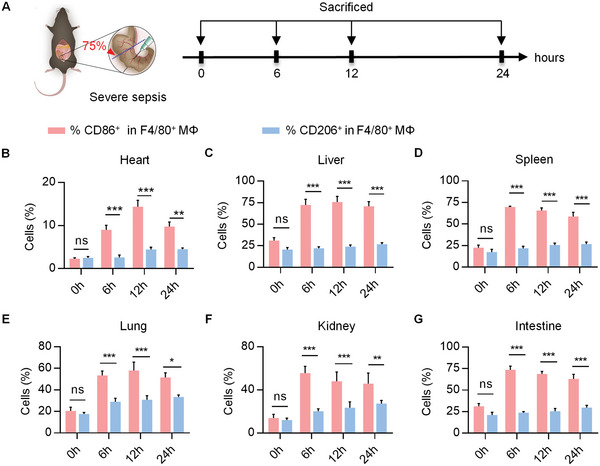
Imbalanced M1/M2 polarization in murine sepsis model. A) Experimental procedures for the septic mouse model and schematic timeline of sample harvest. B–G) Flow cytometry assessment of proinflammatory macrophages (F4/80^+^CD86^+^) and anti‐inflammatory macrophages (F4/80^+^CD206^+^) population in interested tissues of septic mice at 0, 6, 12, and 24 h after CLP. Data are presented as means ± S.E.M. of 3 biological replicates. *p < 0.05, **p < 0.01, ***p < 0.001 by two‐way ANOVA with Sidak's multiple comparison test.

Cecal ligation and puncture (CLP) has become the most widely used model for experimental sepsis.^[^ ^24^, ^25^ ^]^ To investigate the phenotypic changes of macrophages in sepsis, mice were performed the ligation of 75% of the cecum, which means high‐grade sepsis with 100% lethality.^[^ ^24^ ^]^ After the CLP treatment, macrophage polarization in indicated tissues was detected at each time point (**Figure** **1**A). CLP surgery resulted in explosive and continuous expansion of inflammatory macrophages in all tissues (F4/80^+^CD86^+^) (Figure 1B–G, and Figure S1, Supporting Information). Correspondingly, the proinflammatory cytokines (*Tnfα*, *Nos2*, *Il1β*, and *Il6*) in all organs were also increased significantly (Figure S2, Supporting Information), indicating the hyperactivation of proinflammatory macrophages during the sepsis. Of note, there was only a slight increase in the percentage of anti‐inflammatory macrophages (F4/80^+^CD206^+^) (Figure 1B–G, and Figure S1, Supporting Information) and anti‐inflammatory cytokines (*Arg1*, *Mrc1*, *Ym1*, and *Il10*) in each tissue (Figure S2, Supporting Information). Collectively, these results suggested that there was an imbalance of macrophage M1/M2 polarization of sepsis, which is consistent with previous findings.^[^ ^5^, ^26^ ^]^

### 2.2 Excessive mtROS Production Leads to M1 Macrophage Polarization

**Figure 2:**
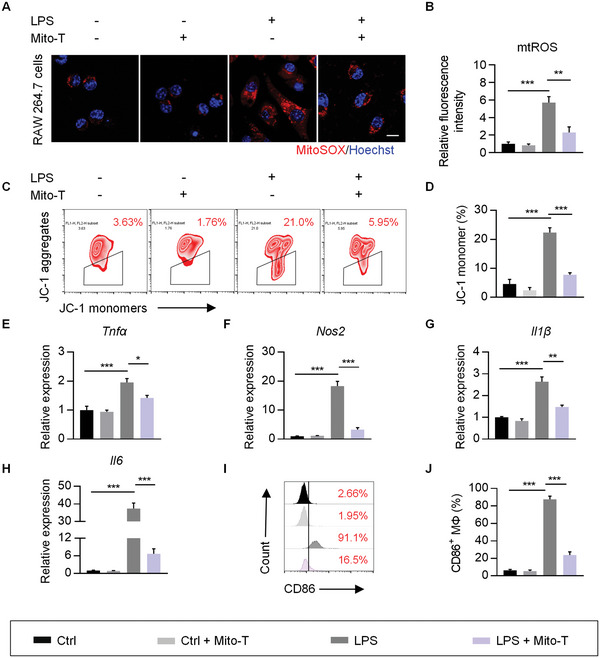
Excessive mtROS promote M1 macrophage polarization. A) Representative confocal images of MitoSOX staining in macrophages. Scale bar, 10 µm. B) Quantitative fluorescence intensity as analyzed by ImageJ software. C) Δ*ψ*m was measured using JC‐1 probe by flow cytometry. The trapeziums show the percentage of cells with decreased Δ*ψ*m. D) Statistical analysis of JC‐1 monomer ratio in RAW 264.7 cells. E–H) qRT‐PCR analysis of pro‐inflammatory cytokine (*Tnfα*, Nos2, Il1*β*, and *Il6*) in RAW 264.7 cells. I) Representative flow cytometry analysis of CD86^+^ macrophages. J) Quantification of CD86^+^ cells. Data are expressed as means ± S.E.M. of 3 biological replicates. *p < 0.05, **p < 0.01, ***p < 0.001 by one‐way ANOVA with Tukey's post hoc test (B, D, E–H, and J).

Overactivation of M1 macrophage is affected by impaired mitochondrial function and excessive mtROS.^[^ ^16^ ^]^ To explore whether mtROS were involved in overactivated M1 in sepsis, we then stimulated RAW 264.7 cells with LPS (lipopolysaccharide) and examined mtROS using fluorescent dyes MitoSOX. The results showed that mtROS were markedly increased in LPS treated macrophages (**Figure** **2**A,B). Mitochondrial membrane potential (ΔΨm) evaluation by JC‐1 staining revealed that LPS treatment decreased the ΔΨm (Figure 2C,D). Meanwhile, the expression of inflammatory cytokines (Figure 2E–H) and the percentage of CD86^+^ subpopulation (Figure 2I,J) was substantially promoted, indicating a promoting effect of LPS on polarization of proinflammatory macrophages. To further determine the effect of mtROS on macrophage polarization, LPS macrophages were treated with mtROS inhibitors (Mito‐TEMPO, Mito‐T). As expected, ΔΨm was recovered and M1 macrophage polarization was inhibited by Mito‐TEMPO (Figure 2A–J). Collectively, these results suggested that LPS exposure led to overproduction of mtROS, which in turn promoted M1 polarization.

### 2.3 Dysregulated Expression of mt‐circRNAs in Macrophages of Septic Mice

**Figure 3:**
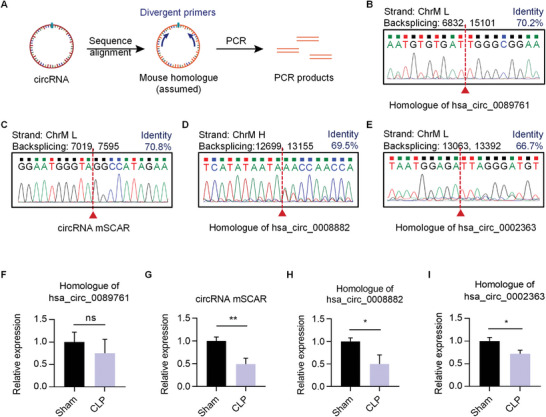
Expression profile of mt‐circRNAs in macrophages of septic mice. A) Design of the divergent PCR primers that specifically amplify the circRNAs. B–E) Sequencing of PCR products from divergent primers, confirming the backspliced junction of circRNAs. The identity of the circRNA between human and mouse is indicated in blue. F–I) Expression of circRNAs in monocytes isolated from sham and septic mice was examined by qRT‐PCR. Data are expressed as means ± S.E.M. of 3 biological replicates. *p < 0.05, **p < 0.01, ***p < 0.001 by Student's *t* test.

There has been a growing interest in mitochondrial circRNAs for their important roles in mitochondria function.^[^ ^19^, ^27^, ^28^ ^]^ At present, there are five mt‐circRNAs (hsa_circ_0089761, circRNA SCAR, hsa_circ_0089763, hsa_circ_0008882, and hsa_circ_0002363) have been reported. We hypothesized that homologues of mt‐circRNAs mentioned above are existed in mouse. Thus, we designed the divergent primers of mouse homologues based on the sequence alignment (**Figure** **3**A), and that homologues of hsa_circ_0089761, circRNA SCAR, hsa_circ_0008882, and hsa_circ_0002363 were detected successfully. The backspliced junction sites were further confirmed by sequencing, which proved that these four candidates were exactly circular RNAs. Among these four circRNAs, homologue of hsa_circ_0008882 is generated from the heavy strand, and others are generated from light strand. And the sequences of these candidates are all highly conserved among human and mouse (Figure 3B–E). We next compared the expression levels of the mt‐circRNAs in monocytes between sham operation mice and septic mice through qRT‐PCR, and we found that the level of circRNA mSCAR, homologue of hsa_circ_0008882, and homologue of hsa_circ_0002363 were downregulated in monocytes of septic mice, and the level of homologue of hsa_circ_0089761 had no significant change (Figure 3F–I).

### 2.4 Construction of Exosome‐Based Nanoplatform for Mitochondrial Delivery of RNA

**Figure 4:**
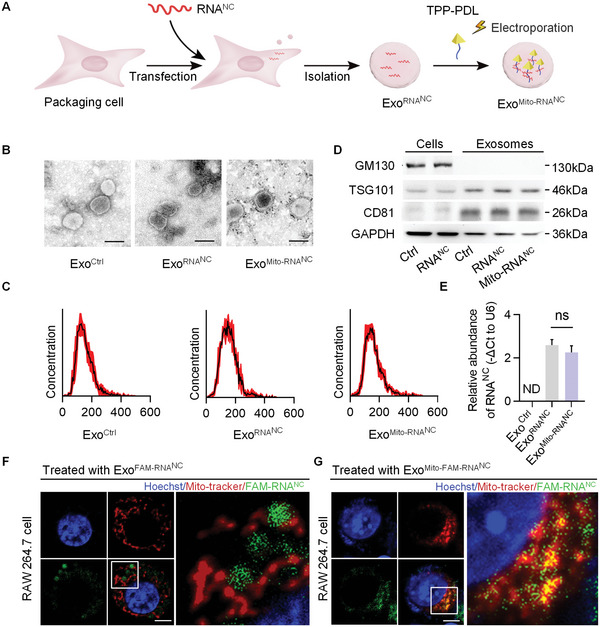
Exosome‐based mitochondrial delivery of RNA. A) The schematic illustration of exosome‐based mitochondria delivery system (Exo^Mito^). B) Representative transmission electron microscope image of indicated functionalized exosomes. Scale bar, 100 nm. C) Size distribution of indicated exosomes analyzed by ZetaView Particle Metrix. D) Western blot analysis of the inclusive and exclusive exosomal markers. Representative image of three different experiments. E) qPCR analysis of the abundance of RNA as determined by the −ΔCt value relative to U6. ND, not determined as Ct value greater than 38. F,G) Representative confocal fluorescence microscopy images. Exo or Exo^Mito^ loaded with FAM‐labeled RNA^NC^ were added into RAW264.7 cells. The mitochondria were stained with MitoTracker (red), and nuclei were stained with Hoechst (blue). Scale bar, 5 µm.

Exosomes can effectively evade detection due to the presence of surface molecules such as CDCK2, CD59, CD55, and CD46, making them ideal drug carriers.^[^ ^29^, ^30^ ^]^ Thus, we constructed an exosome‐based mitochondrial delivery system (named Exo^Mito^ thereafter) to investigate the role of mt‐circRNAs in macrophage polarization and sepsis. The fabrication procedure of Exo^Mito^ is summarized in **Figure** **4**A. Briefly, RNA^NC^ was transfected into HEK293T cells and then passively loaded into intraluminal vesicles (ILVs) through inward budding of the membrane of early endosomes. The secreted exosomes thus were enriched in RNA^NC^. Previous study has found that cells have different immunological responses to chiral molecules.^[^ ^31^ ^]^ Then, cytotoxicity poly‐d‐lysine (PDL) and poly‐l‐lysine (PLL) were compared by CCK‐8 assay. And the IC_50_ values of TPP, PLL, and PDL on RAW 264.7 cells were 212.4, 4.27, and 696.8 µm, respectively (Figure S3A–C, Supporting Information), which means that PDL was safer than PLL. In addition, immunoprecipitation assay confirmed that PDL interacted with RNA with high affinity (Figure S3D, Supporting Information). PDL were selected as nucleic acid adsorption elements and conjugated with TPP group (Figure S3E, Supporting Information), and CCK‐8 assay showed IC_50_ value of TPP‐PDL was 206.6 µm, and TPP‐PDL at 1 µm had minimal effects on cell survival (Figure S3F, Supporting Information). In order to deliver the RNA^NC^ into mitochondria, TPP‐PDL was thus electroporated into the exosomes. TPP‐PDL (1 µm) was loaded into Exo^RNANC^ by electroporation, named Exo^Mito‐RNANC^. To characterize the exosomes, transmission electron microscopy and nanoparticle tracking analysis were used. The results showed that exosomes loaded with TPP‐PDL and RNA were physically similarly to Exo^Ctrl^, with a size diameter ranging between 40 and 160 nm (Figure 4B,C). Further analysis of the exosomal inclusive markers (CD81 and TSG101) and exclusive marker (GM130) by western blot assay additionally confirmed that loading of TPP‐PDL and RNA^NC^ did not change the characteristics of the exosomes (Figure 4D). Finally, we detected the level of RNA^NC^ in Exo^Mito^. As expected, qRT‐PCR revealed that target RNA^NC^ was efficiently loaded in the Exo^Mito^ (Figure 4E). The CCK‐8 assay didn't show any cytotoxicity of Exo^Mito‐RNANC^ on RAW 264.7 cells (Figure S3G, Supporting Information).

Studies have shown that exosomes can be taken up through phagocytosis, micropinocytosis, and endocytosis by recipient cells. Theoretically, the TPP/circRNA complex in the exosomes would be delivered into the mitochondria as TPP is a strong mitochondrial targeting moiety.^[^ ^32^ ^]^ Thus, we examined whether target RNA can be efficiently delivered into mitochondria of macrophages. In order to confirm the mitochondria targeting of TPP‐PDL, fluorescein isothiocyanate (FITC)‐conjugated TPP‐PDL (FITC‐TPP‐PDL) was synthesized (Figure S4A, Supporting Information) and loaded into exosomes as described above, with exosomes loaded with FITC‐PDL served as a control. RAW 264.7 cells were treated with Exo^FITC‐Mito^, and the entry of FITC‐TPP‐PDL into macrophage mitochondria was observed by confocal microscopy. Compared with that in Exo^FITC‐PDL^, there was strong FITC signal observed in the mitochondria in Exo^FITC‐Mito^ treated cells (Figure S4B,C, Supporting Information), suggesting that TPP is indispensable for the targeting of mitochondria. To further confirm that RNA can be delivered into mitochondria by Exo^Mito^, RNA^NC^ labeled with FAM were encapsulated in Exo^Mito^ and incubated with RAW 264.7 cells. As expected, robust localization of FAM‐labeled RNA^NC^ was observed in mitochondria, whereas RNA^NC^ was randomly distributed in the cells when there was no TPP (Figure 4F,G). Together, these data suggested that this mitochondria delivery system can effectively deliver RNA^NC^ into mitochondria. Compared with the synthetic materials used for mitochondrial drug delivery,^[^ ^33^ ^]^ the exosome‐based system we proposed here have super advantage in term of immune response.^[^ ^34^, ^35^ ^]^

### 2.5 Exosome‐Based Delivery of circRNA mSCAR Orchestrates Macrophage Activation

To investigate the effect of mt‐circRNAs on mitochondria function and macrophage polarization, we encapsulated mt‐circRNAs into exosomes flowed by electroporation with TPP‐PDL (Figure S5A,B, Supporting Information), with the resultant exosomes named Exo^Mito‐circRNA^. Absolute quantification qPCR confirmed that mt‐circRNAs could be encapsulated into exosomes, with 2.14 ± 0.21 copies of circRNA mSCAR, 2.51 ± 0.50 copies of homologue of hsa_circ_0008882, and 2.10 ± 0.16 copies of homologue of hsa_circ_0002363 per exosome, respectively. RNase R exonuclease treatment further confirmed that these mt‐circRNAs encapsulated were exactly the circular structure (Figure S5C–H, Supporting Information). Macrophages treated with Exo^Mito‐circRNA^ had much higher levels of RNase R resistant mt‐circRNAs in the mitochondria, as observed from qPCR analysis of the mitochondria isolated from macrophages (Figure S5I–K, Supporting Information).

**Figure 5:**
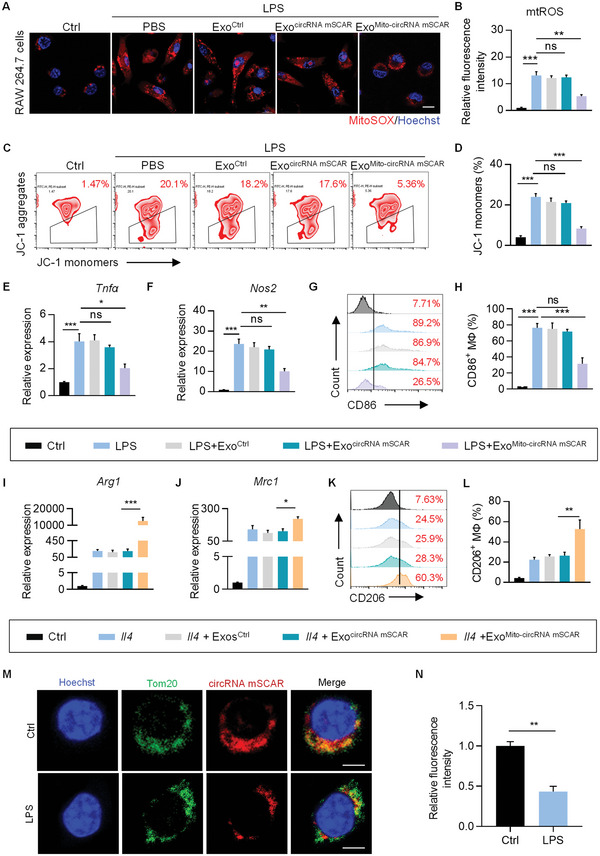
Exosome‐based delivery of circRNA mSCAR orchestrates macrophage activation. A) Representative confocal images of MitoSOX in macrophages treated as indicated. Scale bar, 10 µm. B) Quantitative fluorescence intensity as analyzed by ImageJ software. C) Δ*ψ*m was measured using JC‐1 probe by flow cytometry. The trapeziums show the percentage of cells with decreased Δ*ψ*m. D) Statistical analysis on JC‐1 monomer ratio in RAW 264.7 cells. E,F) Levels of proinflammatory cytokines (*Tnfα* and *Nos2*) in RAW 264.7 cells were examined by qRT‐PCR. G) Representative flow cytometry analysis of CD86^+^ macrophages. H) Quantification of CD86^+^ cells. I,J) Levels of anti‐inflammatory cytokines (*Arg1* and *Mrc1*) in RAW 264.7 cells were examined by qRT‐PCR. K) Representative flow cytometry analysis of CD206^+^ macrophages. L) Quantification of CD206^+^ cells. M) The FISH analysis for endogenous circRNA mSCAR and co‐immunostaining of Tom20 in macrophages treated as indicated. Scale bar, 5 µm. N) Quantitative fluorescence intensity as analyzed by Image J. Data are expressed as means ± S.E.M. of 3 biological replicates. *p < 0.05, **p < 0.01, ***p < 0.001 by one‐way ANOVA with Tukey's post hoc test or Student's *t* test.

Then we treated the LPS‐stimulated macrophages with Exo^Ctrl^, Exo^circRNA mSCAR^, and Exo^Mito‐circRNA mSCAR^, respectively, and found that mitochondria‐specific delivery of circRNA mSCAR reduced the mtROS and reversed depression of ΔΨm in LPS‐stimulated macrophages (**Figure** **5**A–D), which demonstrating a strong effect of circRNA mSCAR in mitochondria function. To ask whether circRNA mSCAR affects the macrophage polarization, flow cytometry was used to detect macrophage phenotypes. As expected, mitochondrial delivery of circRNA mSCAR substantially attenuated the level of inflammatory cytokines (Figure 5E,F and Figure S6A,B, Supporting Information) and CD86^+^ percentage (Figure 5G,H, Supporting Information), indicating an inhibiting effect of circRNA mSCAR on polarization of proinflammatory macrophages. In order to observe effect of circRNA mSCAR on anti‐inflammatory macrophage activation, RAW 264.7 cells were treated with *Il4* and co‐cultured with Exo^Ctrl^, Exo^circRNA mSCAR^, and Exo^Mito‐circRNA mSCAR^, respectively. Results shown that circRNA mSCAR can promote the level of anti‐inflammatory cytokines (Figure 5I,J and Figure S6C,D, Supporting Information) and CD206^+^ percentage (Figure 5K,L), further suggesting the therapeutic benefit of circRNA mSCAR in inflammatory disease. In contrast, Exo^Mito^ encapsulated with homologue of hsa_circ_0008882 and homologue of hsa_circ_0002363 didn't show significant effects on either mtROS or macrophage polarization in RAW264.7 cells upon LPS stimulation (Figure S7, Supporting Information). Next, to further confirm the mitochondrial localization of circRNA mSCAR in macrophages, we performed fluorescence in situ hybridization (FISH) using a specific probe to circRNA mSCAR. As expected, circRNA mSCAR was predominantly localized in mitochondria, and the level of circRNA mSCAR was downregulated in LPS‐stimulated macrophages (Figure 5M,N). Together, these data suggested that circRNA mSCAR, rather than other mitochondrial circRNAs orchestrates macrophage polarization by regulating the level of mtROS. Notably, both linear and circular RNA could be transcribed from the same gene locus. For example, Zhao et al. has shown that MT‐LIPCAR (JA760602) is known linear transcript of circRNA SCAR, which is abnormally expressed in disease models.^[^ ^36^ ^]^ Thus, the physiological and therapeutic function of linear version cannot be ignored.

### 2.6 Exosome‐Based Delivery of circRNA mSCAR Alleviates Sepsis in Mouse Model

**Figure 6:**
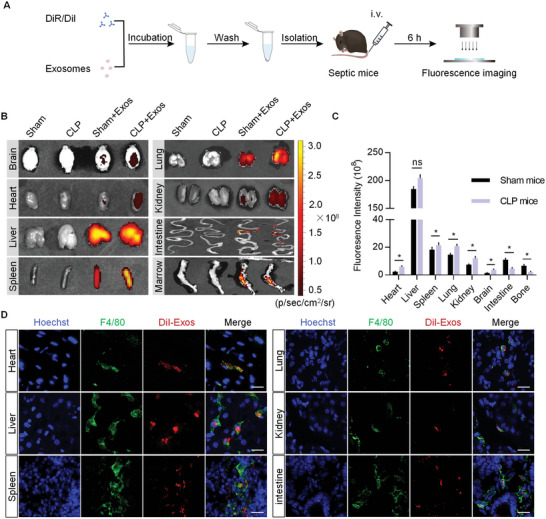
Exosomes can be effectively taken up by macrophages in septic mouse model. A) Schematic diagram of the experimental procedure. Mice were injected with DiR/DiI‐labeled exosomes (4 µg g^−1^) via tail vein and the distribution of the exosomes were then monitored 6 h later. B) In vivo fluorescence imaging analysis of the distribution of the DiR‐labeled exosomes in different organs, including brain, heart, liver, spleen, lung, kidney, intestine, and bone marrow. C) Quantification of the fluorescence signal intensity. Data are expressed as means ± S.E.M. (*n* = 4). *p < 0.05 by Student's *t* test. D) Representative fluorescence microscopic images of DiI‐labeled (red) exosomes uptake into macrophages (F4/80, green) in indicated organs. The nuclei were counter‐stained with Hoechst (blue). Representative images of at least three mice. Scale bar, 20 µm.

In the following experiment, we investigated the therapeutic effects of Exo^Mito‐circRNA mSCAR^ in septic mice. To profile the in vivo distribution of the exosomes, DiR‐labeled exosomes were tracked in sham and septic mice (**Figure** **6**A). In vivo imaging system demonstrated that systemically administered exosomes were mainly localized in liver and spleen of both sham operation mice and septic mice, which are the major organs in the mononuclear phagocyte system. Notably, a substantial number of exosomes were accumulated in other organs like heart, lung, kidney of septic mice (Figure 6B,C), which could be explained by the accumulated immune cells in the septic mice and would be also beneficial for the treatment of sepsis. In contrast, exosomes distributed to the intestine and bone marrow was reduced in septic mice models (Figure 6B,C), which may be attributed to macrophage mobilization in sepsis. To further confirm the exosome uptake by macrophages in various organs of mice, DiI‐labeled exosomes were injected into septic mice via tail vein (Figure 6A) and co‐localization of exosomes with the macrophage marker F4/80 in inflamed tissues was observed by confocal fluorescence microscopy (Figure 6D). It shouldn't be ignored that a small amount of exosomes might be also internalized by other phagocytes (e.g., neutrophile) or/and other parenchymal cells. Since it is well established that over‐produced mtROS might be also occurred in other cell types in the context of sepsis,^[^ ^37^, ^38^ ^]^ exosomes delivered into these cells may also play a therapeutic role in sepsis.

**Figure 7:**
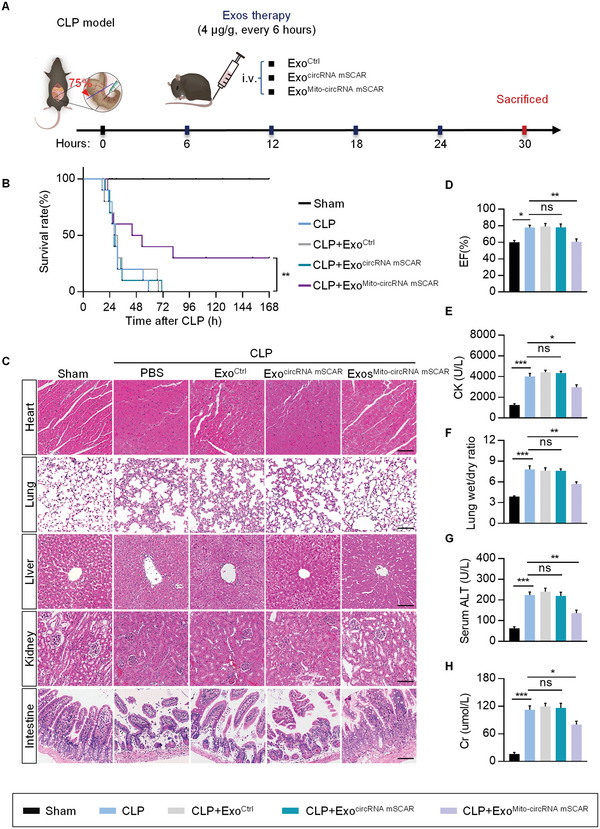
Exosome‐based delivery of circRNA mSCAR alleviates sepsis in mouse model. A) Schematic diagram of the experimental procedure. Mice were treated with i.v. injections of Exo^Ctrl^, Exo^circRNA mSCAR^, Exo^Mito‐circRNA mSCAR^ at 6, 12, 18, and 24 h after CLP. Mice receiving sham operation were used as control. B) Survival analysis of the Sham and CLP mice with indicated treatments (*n* = 10). **p < 0.01 by log‐rank test. C) Representative H&E staining images for tissue sections from the sham and septic mice with Exo^Ctrl^, Exo^circRNA mSCAR^, or Exo^Mito‐circRNA mSCAR^ treatments. *n* = 6 mice for each group. Scale bar, 100 µm. D) Ejection fraction (EF) was measured from mice in various groups. E–H) Blood biochemistry analysis of CK (heart function), ALT (liver function), and Cr (kidney function). Lung function was demonstrated by wet/dry ratio. Data are expressed as means ± S.E.M. (*n* = 6). *p < 0.05, **p < 0.01, ***p < 0.001 by one‐way ANOVA with Tukey's post hoc test.

To further evaluate the therapeutic efficacy of Exo^Mito‐circRNA mSCAR^, septic mice were given treatments with Exo^Ctrl^, Exo^circRNA mSCAR^, and Exo^Mito‐circRNA mSCAR^, respectively (**Figure** **7**A). qRT‐PCR analysis revealed that the proinflammatory cytokines, such as *Tnfα*, *Nos2*, *Il1β*, and *Il6*, were reduced after Exo^Mito‐circRNA mSCAR^ treatment. In addition, the level of anti‐inflammatory factors, such as *Arg1*, *Mrc1*, *Ym1*, and *Il10*, were increased (Figure S8A–F, Supporting Information). Consistent with the inhibited inflammation by Exo^Mito‐circRNA mSCAR^, Exo^Mito‐circRNA mSCAR^ treatment prolonged the survival of septic mice (Figure 7B).

Mortality in patients with sepsis is correlated with the multiple organ failures.^[^ ^4^ ^]^ Consistently, in the sepsis mice, abnormal arrangement of cardiomyocytes, and notable edema of myocardial cells. In the lungs of septic mice, alveolar damages, edema, and interstitial thickening were observed. Liver in sepsis also showed loss of hepatic sinusoids and diffusive cell death. In addition, tubular cell swelling, hemorrhage, and tubular dilatation was observed in kidney of sepsis. Disordered and ruptured intestinal villi were also observed in septic mice (Figure 7C). Treatment of Exo^Mito‐circRNA mSCAR^ attenuated all these histological changes in the above organs (Figure 7C). In addition, the abnormal systolic function and diastolic function, were also rescued by Exo^Mito‐circRNA mSCAR^, as seen from the ejection fraction (EF) (Figure 7D) and E/A ratio (Figure S9, Supporting Information) in septic mice. Similarly, the increased creatinine (CK), lung wet/dry ratio, and creatine (Cr) as well as alanine transaminase (ALT) in sepsis were also reduced after Exo^Mito‐circRNA mSCAR^ treatment (Figure 7E–H). Based on the evidence observed above, we concluded that Exos^Mito‐circRNA mSCAR^ could attenuate sepsis effectively.

## 3 Conclusion

In summary, we here uncovered for the first time that downregulated circRNA mSCAR promotes the development of sepsis. Exosomes encapsulated with the therapeutic circRNA mSCAR followed by TPP‐PDL electroporation, can achieve mitochondrial delivery of circRNA mSCAR. Precise delivery of circRNA mSCAR into mitochondrial could robustly reverse M1 polarization through reducing mtROS, and thus improve the outcome of septic condition, emerging as a promising intervening strategy of sepsis and other inflammatory diseases. As linear version of circRNA mSCAR is co‐transcribed from mtDNA and by the constructed plasmid, the linear version might be also involved, which needs further studies.

## Conflict of Interest

The authors declare no conflict of interest.

## Author Contributions

Y.X., M.W., and Y.Z. conceived and supervised the work. L.F., L.Y., Z.L., Z.W., and W.S. designed and performed experiments, and analyzed data. S.Q. and W.Z. assisted in the exosome isolation. D.X., L.Q., and G.Y revised the manuscript.

## Supporting information

Supporting Information

## Data Availability

The data that support the findings of this study are available in the supplementary material of this article.

## References

[1] C. Rhee , S. Gohil , M. Klompas , N. Engl. J. Med. 2014, 370, 1673. 24738642 10.1056/NEJMp1400276 PMC4718398

[2] M. Singer , C. S. Deutschman , C. W. Seymour , M. Shankar‐Hari , D. Annane , M. Bauer , R. Bellomo , G. R. Bernard , J. D. Chiche , C. M. Coopersmith , R. S. Hotchkiss , M. M. Levy , J. C. Marshall , G. S. Martin , S. M. Opal , G. D. Rubenfeld , T. van der Poll , J. L. Vincent , D. C. Angus , JAMA, J. Am. Med. Assoc. 2016, 315, 801. 10.1001/jama.2016.0287 PMC4968574 26903338

[3] T. van der Poll , S. J. van Deventer , Infect. Dis. Clin. North Am. 1999, 13, 413. 10340175 10.1016/s0891-5520(05)70083-0

[4] C. Lelubre , J. L. Vincent , Nat. Rev. Nephrol. 2018, 14, 417. 29691495 10.1038/s41581-018-0005-7

[5] M. Bosmann , P. A. Ward , Trends Immunol. 2013, 34, 129. 23036432 10.1016/j.it.2012.09.004 PMC3543471

[6] B. E. Ragle , J. Bubeck Wardenburg , Infect. Immun. 2009, 77, 2712. 19380475 10.1128/IAI.00115-09 PMC2708543

[7] S. H. Gregory , W. H. Chen , S. Mott , J. E. Palardy , N. A. Parejo , S. Heninger , C. A. Anderson , A. W. Artenstein , S. M. Opal , A. S. Cross , Vaccine 2010, 28, 2908. 20170768 10.1016/j.vaccine.2010.01.067 PMC2847053

[8] D. N. Cruz , M. Antonelli , R. Fumagalli , F. Foltran , N. Brienza , A. Donati , V. Malcangi , F. Petrini , G. Volta , F. M. Bobbio Pallavicini , F. Rottoli , F. Giunta , C. Ronco , JAMA, J. Am. Med. Assoc. 2009, 301, 2445. 10.1001/jama.2009.856 19531784

[9] U. Andersson , K. J. Tracey , Annu. Rev. Immunol. 2011, 29, 139. 21219181 10.1146/annurev-immunol-030409-101323 PMC4536551

[10] K. Singh , L. X. Zhang , K. Bendelja , R. Heath , S. Murphy , S. Sharma , J. F. Padbury , Y. P. Lim , Pediatr. Res. 2010, 68, 242. 20520583 10.1203/PDR.0b013e3181e9fdf0 PMC2928396

[11] T. van der Poll , F. L. van de Veerdonk , B. P. Scicluna , M. G. Netea , Nat. Rev. Immunol. 2017, 17, 407. 28436424 10.1038/nri.2017.36

[12] L. C. Davies , S. J. Jenkins , J. E. Allen , P. R. Taylor , Nat. Immunol. 2013, 14, 986. 24048120 10.1038/ni.2705 PMC4045180

[13] M. Valko , D. Leibfritz , J. Moncol , M. T. Cronin , M. Mazur , J. Telser , Int. J. Biochem. Cell Biol. 2007, 39, 44. 16978905 10.1016/j.biocel.2006.07.001

[14] T. A. Wynn , L. Barron , Semin. Liver Dis. 2010, 30, 245. 20665377 10.1055/s-0030-1255354 PMC2924662

[15] D. J. Puleston , M. D. Buck , R. I. Klein Geltink , R. L. Kyle , G. Caputa , D. O'Sullivan , A. M. Cameron , A. Castoldi , Y. Musa , A. M. Kabat , Y. Zhang , L. J. Flachsmann , C. S. Field , A. E. Patterson , S. Scherer , F. Alfei , F. Baixauli , S. K. Austin , B. Kelly , M. Matsushita , J. D. Curtis , K. M. Grzes , M. Villa , M. Corrado , D. E. Sanin , J. Qiu , N. Pallman , K. Paz , M. E. Maccari , B. R. Blazar , et al., Cell Metab. 2019, 30, 352. 31130465 10.1016/j.cmet.2019.05.003 PMC6688828

[16] E. L. Mills , B. Kelly , A. Logan , A. S. H. Costa , M. Varma , C. E. Bryant , P. Tourlomousis , J. H. M. Dabritz , E. Gottlieb , I. Latorre , S. C. Corr , G. McManus , D. Ryan , H. T. Jacobs , M. Szibor , R. J. Xavier , T. Braun , C. Frezza , M. P. Murphy , L. A. O'Neill , Cell 2016, 167, 457. 27667687 10.1016/j.cell.2016.08.064 PMC5863951

[17] J. Li , B. Diao , S. Guo , X. Huang , C. Yang , Z. Feng , W. Yan , Q. Ning , L. Zheng , Y. Chen , Y. Wu , Nat. Commun. 2017, 8, 1322. 29109438 10.1038/s41467-017-01327-4 PMC5673889

[18] W. R. Jeck , J. A. Sorrentino , K. Wang , M. K. Slevin , C. E. Burd , J. Liu , W. F. Marzluff , N. E. Sharpless , RNA 2013, 19, 141. 23249747 10.1261/rna.035667.112 PMC3543092

[19] X. Liu , X. Wang , J. Li , S. Hu , Y. Deng , H. Yin , X. Bao , Q. C. Zhang , G. Wang , B. Wang , Q. Shi , G. Shan , Sci China: Life Sci. 2020, 63, 1429. 32048164 10.1007/s11427-020-1631-9

[20] X. Liu , G. Shan , Front. Cell Dev. Biol. 2021, 9, 713729. 34395442 10.3389/fcell.2021.713729 PMC8362354

[21] S. Memczak , M. Jens , A. Elefsinioti , F. Torti , J. Krueger , A. Rybak , L. Maier , S. D. Mackowiak , L. H. Gregersen , M. Munschauer , A. Loewer , U. Ziebold , M. Landthaler , C. Kocks , F. le Noble , N. Rajewsky , Nature 2013, 495, 333. 23446348 10.1038/nature11928

[22] Q. Zheng , C. Bao , W. Guo , S. Li , J. Chen , B. Chen , Y. Luo , D. Lyu , Y. Li , G. Shi , L. Liang , J. Gu , X. He , S. Huang , Nat. Commun. 2016, 7, 11215. 27050392 10.1038/ncomms11215 PMC4823868

[23] R. A. Smith , R. C. Hartley , H. M. Cochemé , M. P. Murphy , Trends Pharmacol. Sci. 2012, 33, 341. 22521106 10.1016/j.tips.2012.03.010

[24] D. Rittirsch , M. S. Huber‐Lang , M. A. Flierl , P. A. Ward , Nat. Protoc. 2009, 4, 31. 19131954 10.1038/nprot.2008.214 PMC2754226

[25] J. A. Buras , B. Holzmann , M. Sitkovsky , Nat. Rev. Drug Discov. 2005, 4, 854. 16224456 10.1038/nrd1854

[26] C. Nathan , Nature 2002, 420, 846. 12490957 10.1038/nature01320

[27] Q. Zhao , J. Liu , H. Deng , R. Ma , J. Y. Liao , H. Liang , J. Hu , J. Li , Z. Guo , J. Cai , X. Xu , Z. Gao , S. Su , Cell 2020, 183, 76. 32931733 10.1016/j.cell.2020.08.009

[28] Z. Wu , H. Sun , C. Wang , W. Liu , M. Liu , Y. Zhu , W. Xu , H. Jin , J. Li , Mol. Ther. – Nucleic Acids 2020, 20, 801. 32438315 10.1016/j.omtn.2020.04.017 PMC7240210

[29] M. J. Haney , N. L. Klyachko , Y. Zhao , R. Gupta , E. G. Plotnikova , Z. He , T. Patel , A. Piroyan , M. Sokolsky , A. V. Kabanov , E. V. Batrakova , J. Controlled Release 2015, 207, 18. 10.1016/j.jconrel.2015.03.033 PMC4430381 25836593

[30] X. Zhu , M. Badawi , S. Pomeroy , D. S. Sutaria , Z. Xie , A. Baek , J. Jiang , O. A. Elgamal , X. Mo , K. Perle , J. Chalmers , T. D. Schmittgen , M. A. Phelps , J. Extracell. Vesicles 2017, 6, 1324730. 28717420 10.1080/20013078.2017.1324730 PMC5505007

[31] X. Zhao , S. Q. Zang , X. Chen , Chem. Soc. Rev. 2020, 49, 2481. 32176233 10.1039/d0cs00093k

[32] J. Zielonka , J. Joseph , A. Sikora , M. Hardy , O. Ouari , J. Vasquez‐Vivar , G. Cheng , M. Lopez , B. Kalyanaraman , Chem. Rev. 2017, 117, 10043. 28654243 10.1021/acs.chemrev.7b00042 PMC5611849

[33] Q. Peng , S. Zhang , Q. Yang , T. Zhang , X. Q. Wei , L. Jiang , C. L. Zhang , Q. M. Chen , Z. R. Zhang , Y. F. Lin , Biomaterials 2013, 34, 8521. 23932500 10.1016/j.biomaterials.2013.07.102

[34] M. Cully , Nat. Rev. Drug Discov. 2021, 20, 6. 33907314 10.1038/d41573-021-00071-1

[35] P. H. L. Tran , D. Xiang , T. T. D. Tran , W. Yin , Y. Zhang , L. Kong , K. Chen , M. Sun , Y. Li , Y. Hou , Y. Zhu , W. Duan , Adv. Mater. 2020, 32, 1904040. 10.1002/adma.201904040 31531916

[36] Q. Zhao , J. Liu , H. Deng , R. Ma , J. Y. Liao , H. Liang , J. Hu , J. Li , Z. Guo , J. Cai , X. Xu , Z. Gao , S. Su , Cell 2020, 183, 76. 32931733 10.1016/j.cell.2020.08.009

[37] D. B. Zorov , M. Juhaszova , S. J. Sollott , Physiol. Rev. 2014, 94, 909. 24987008 10.1152/physrev.00026.2013 PMC4101632

[38] G. Cepinskas , J. X. Wilson , J. Clin. Biochem. Nutr. 2008, 42, 175. 18545638 10.3164/jcbn.2008026 PMC2386519

