# Supplementary material for: Exosome‐Based Mitochondrial Delivery of circRNA mSCAR Alleviates Sepsis by Orchestrating Macrophage Activation
Source: Adv Sci (Weinh). 2023 Mar 25;10(14):2205692. doi: 10.1002/advs.202205692 (PMC10190648; doi:10.1002/advs.202205692)
Supplement: Supplementary file 1 — Supporting Information [file ADVS-10-2205692-s001.pdf]

Supporting Information

Exosome-based mitochondrial delivery of circRNA mSCAR alleviates sepsis by orchestrating macrophage activation

Li Fan[1,#], Li Yao[2,#], Zhelong Li[3,#], Zhuo Wan[4], Wenqi Sun[3], Shuo Qiu[3], Wei Zhang[5], Dan Xiao[1], Liqiang Song[6], Guodong Yang[7], Yi Zhang[8,], Mengying Wei[7,*], Xuekang Yang[1,*]*

[1] Department of Burns and Cutaneous Surgery, Xijing Hospital, Fourth Military Medical University, Xi'an, 710032, People's Republic of China.

[2] Department of Pathology, Xi'an No. 3 Hospital, The Affiliated Hospital of Northwest University, Xi'an, 710018, People's Republic of China.

[3] Department of Ultrasound Diagnostics, Tangdu Hospital, Fourth Military Medical University, Xi'an, 710038, People's Republic of China.

[4] Department of Hematology, Tangdu Hospital, Fourth Military Medical University, Xi'an, 710038, People's Republic of China.

[5] Department of Respiratory Medicine, Tangdu Hospital, Fourth Military Medical University, Xi'an, 710038, People's Republic of China.

[6] Department of pulmonary and critical care medicine, Xijing Hospital, Fourth Military Medical University, Xi'an, 710032, People's Republic of China.

[7] The State Laboratory of Cancer Biology, Department of Biochemistry and Molecular Biology, Fourth Military Medical University, Xi'an, 710032, People's Republic of China.

[8] Department of Dental Clinical Diagnostics, School of Stomatology, Fourth Military Medical University, Xi'an, 710032, People's Republic of China.

[#]These authors contributed equally to this work.

[*]Corresponding authors.

Email: zhangyi@fmmu.edu.cn, weimengy@fmmu.edu.cn, sskzzb06@fmmu.edu.cn.

Experimental Section

Mice: Male C57BL/6 mice (8-10 weeks old, 22-25 g) were used. Mice were maintained under specific pathogen free conditions. The animal experimental procedures were performed strictly following the guidelines approved by the Institutional Animal Experiment Administration Committee of the Fourth Military Medical University. All experiments were authorized by the Animal Care and Ethic Committee of Fourth Military Medical University (Approval NO. KY20213144-1).

Cecal ligation and puncture surgery: High-grade sepsis model was performed as described [1]. Mice were anesthetized through intraperitoneal injection of pentobarbital sodium (1%). After anesthesia, the peritoneum was opened under sterile condition, and 75% of the cecum was tightly ligated using 4.0-silk suture and punctured once with a 21-gauge needle. A small amount of feces was extruded from perforation site. Then, the cecum was returned to peritoneal cavity and the abdominal incision was closed with 4.0-silk suture. In sham-operated group, mice were subjected to the same procedures except for the ligation and perforation of the cecum.

Cell Culture: RAW 264.7 cells and HEK293T cells were obtained from ATCC. HEK293T cells were cultured in DMEM (Dulbecco’s Modified Eagle Medium) medium containing 10% fetal bovine serum (Excell Bio, China) and 1% antibiotics (Solarbio, China). RAW 264.7 macrophages were grown in RPMI (Roswell Park Memorial Institute) medium with 10% FBS and 1% antibiotics. Cells were changed with fresh medium every other day and maintained at 37℃ in 5% CO_2.

Plasmid construction: The overexpression plasmids for circRNA mSCAR, homologues of hsa_circ_0008882, hsa_circ_0002363 were constructed according to previous reports[2]. Briefly, corresponding circRNA cDNAs were amplified by PCR primers flanked with corresponding enzyme sites. The amplicon was digested and cloned into the pLC5-ciR vector. The right clones were then confirmed by DNA sequencing. PCR primers used are listed in Table S1.

Synthesis of TPP-PDL: The mitochondria-targeting peptide TPP-PDL was synthesized by Ruixibio (Xi’an China). Briefly, PDL (100.000 mg, 0.08 mol), TPP (35.200 mg, 0.095 mol), EDC (30.500 mg, 0.159 mol), and DMAP (4.900 mg, 0.040 mol) were dissolved in 1 mL of DMSO. The resultant product was purified by dialysis (MWCO 1000 Da) for 24 h. The final product was collected after lyophilization. For FITC-conjugated TPP-PDL, PDL (500.000 mg, 0.400 mol), FITC (306.000 mg, 0.800 mol) were dissolved in 7 mL of DMSO, followed by TEA (330 μ l) addition. Then, the dissolved TPP (293.000 mg, 0.800 mol), EDC (304.000 mg, 1.580 mol), and DMAP (10.000 mg, 0.082 mol) in 4 ml DMSO, was added dropwise to PDL-FITC suspension. The product was then precipitated and the FITC-conjugated TPP-PDL was further obtained by vacuum drying.

In vitro cytotoxicity analysis: To determine the cytotoxicity of TPP, PLL, PDL, and TPP-PDL, RAW 264.7 cells were seeded in 96-well plates at a density of 8×10^3 cells/well. Cells were treated with various concentrations of TPP, PDL, PLL, and TPP-PDL. Then, cell viability was analyzed by CCK-8 (Elabscience, China) as instructed. The potency of the cytotoxicity effect was expressed as half maximal inhibitory concentration (IC_{50}). The cytotoxicity of indicated exosomes were detected by CCK-8 assay after treating RAW 264.7 cells with indicated exosomes (40 μg/ml).

RNA immunoprecipitation: To explore whether PDL binds RNA, flag-tagged PDL (AP, China) were incubated with RNA^{NC}, followed by precipitation with anti-flag antibody agarose beads (QualitYard, China). Then unbound RNAs were washed out with PBS for three times and the precipitated RNAs were isolated with TRIzol® reagent, followed by detection of the RNA^{NC} abundance by qRT-PCR.

Exosome preparation: HEK293T cells were used as the exosome donor cells in the study. HEK293T cells were transfected with RNA^{NC} (GenePharma, China) or circRNA-expressing vectors by HighGene (ABclonal Technology, China) as indicated. Six hours later, cells were

further cultured in serum-free medium for another 48 h. For the isolation of exosomes, cell culture supernatants were centrifugated at 2,000 × g for 10 min and then 10,000 × g for 30 min to discard cells and cellular debris. Then, the resulting supernatants were filtered through 0.22 μm filter to remove large particles, followed by centrifugation at 100, 000 × g for 70 min. To remove contaminating proteins, the pellet was washed with PBS and the isolation procedure was repeated another time. The isolated exosomes were resuspended in PBS and stored at -80℃.

To load TPP-PDL into exosomes, exosomes were electroporated with 1 μM TPP-PDL in 4 mm wide electroporation cuvettes at 700 V/150 mF and immediately transferred onto ice. Then the exosomes were washed with cold PBS to remove free peptide, followed by centrifugation at 12,000 × g.

Characterization of exosomes: Exo^{Ctrl}, Exo^{RNA}, Exo$^{Mito\text{-}RNA}$ were analyzed by electron microscopy. Briefly, the exosomes were dropped onto the grid before stained with 2% uranyl acetate. Then exosomes were dried for 0.5 h and examined by the electron microscope (JEM-2000 EX TEM, JEOL Ltd., Tokyo, Japan). For size distribution analysis, isolated exosomes were uniformly diluted to 500 ng/mL (at protein concentration), followed by analysis on the Zeta View (Particle Metrix, GER).

The inclusive and exclusive markers in exosomes were verified by Western blotting. Briefly, total protein of samples was prepared with RIPA Lysis Buffer (Beyotime, China) at 4 °C for 30 min. Protein concentration was determined by Pierce BCA Protein Assay Kit (Thermo, US). Subsequently, equal amounts of proteins were concentrated on SDS-PAGE (6%) and separated in SDS-PAGE (12%), and then transferred onto nitrocellulose filter membranes. The nitrocellulose membrane was blocked with 5% bovine serum albumin for 1 h and then incubated with primary antibodies, including anti-GM130 (Abcam, UK), anti-TSG101 (Abcam, UK), anti-CD81 (Abcam, UK), and anti-GAPDH (BBI life sciences, China) overnight at 4 °C. After washing three times in TBST, the membranes were incubated with anti-rabbit (CST, US) or anti-mouse (CST, US) secondary antibodies corresponding to the primary antibodies at room temperature for 1 h and visualized using the ECL Prime Western Blotting Detection Reagent (GE Healthcare, Buckinghamshire UK).

Tracking of mitochondrial delivery of RNA: To evaluate the efficiency of mitochondrial delivery of RNA by Exo^{Mito}, RAW 264.7 cells were cultured in confocal dish and incubated indicated exosomes encapsulated with fluorescence (FITC/FAM) labeled RNA or TPP-PDL for 24 h. Thereafter, the mitochondria were stained with 100 nM MitoTracker^{TM} Red (Invitrogen, US) for 30 min. After being washed with PBS, the nuclei were stained with Hoechst (Invitrogen, US). Subcellular distribution of FITC or FAM signals in the cells were then examined and imaged under a confocal microscope (Nikon A1R, Japan).

Detection of mtROS: Mitochondrial ROS were detected by MitoSOX^{TM} (MK, China). Briefly, cells treated with 100 ng/ml LPS (Sigma, US), 100 μM Mito-TEMPO (Enzo life sciences, China) , or in different combination with indicated intervals, were incubated with 5 μM MitoSOX^{TM} working solution at 37℃ for 20 min in dark. Cells were washed thrice with PBS and counterstained with 1 μg/ml Hoechst. Fluorescent signals were analyzed by Nikon A1 Spectral Confocal Microscope.

Isolation of Mitochondria: Mitochondria of cultured cells was isolated by Mitochondria Isolation Kit (Thermo Fisher Scientific, China). Cells were collected and re-suspended in ice-cold isolation buffer for 15 min. After homogenized by 20 strokes, nuclei were discarded by centrifuged twice at 700×g at 4℃ for 10 min. Mitochondria were sedimented at 12, 000×g at 4℃ for 10 min.

RT-PCR: Total RNA of the exosomes, cells, tissues or mitochondria was extracted using TRIzol® reagent (Invitrogen, USA). Reverse-transcription of target RNAs was performed by

Transcriptor First-strand cDNA Synthesis Kit or miRcute Plus miRNA Synthesis Kit according to manufacturers' instructions. Quantitative PCR was performed by the FastStart Essential DNA Green Master (Indianapolis, IN, US). Relative gene expression was normalized to β-actin or U6 and quantified with the $2^{-\Delta\Delta Ct}$ or -ΔCt method for comparison. The sequences of PCR primers are provided in Table S1. CircRNAs copy numbers in exosomes was measured by absolute RNA quantification according to previous reports [3]. Briefly, standard curve was created by series dilution of purified and quantified PCR products of indicated circRNAs. Total RNA was extracted from exosomes, processed with RNase R, and reversely transcribed into cDNAs. The copy number of circRNA mSCAR was then quantified from standard curve, and then divided by the exosome particle number obtained by NTA.

Flow Cytometry: The interest tissues from septic mice were collected. Single cell suspension was prepared by digestion with collagenase Ⅳ (Gibco, US). Red blood cells were lysed using ACK buffer (Sangon Biotech) and cells were washed with PBS twice. Then the samples resuspended with phosphate-buffered saline supplemented with 0.2% bovine serum albumin, 0.01% NaN_3, were incubated with fluorochrome-conjugated Abs (CD86-FITC, CD206-PE and F4/80-APC, Biolegend, US) for 30 min at 4℃. Cells were then washed twice with flow-cytometry buffer. Fluorescence was quantitated on Beckman CytoFlex as instructed, and the data were analyzed with Flowjo VX software. Flow cytometry analysis of RAW 264.7 polarization was performed in a similar way.

Mitochondrial membrane potential was studied using the JC-1 (MK, China) according to the manufacturer's instructions. Briefly, samples were incubated with 10 μg/mL JC-1 at 37℃ for 20 min and rinsed three times with PBS. Fluorescent signals were analyzed by flow cytometry (Beckman CytoFlex, US).

RNA FISH: RNA FISH assay was performed using RNA FISH kit (GenePharma, China) according to manufacturer's instruction. Briefly, RAW 264.7 cells were fixed, permeabilized, and blocked for 5% bovine serum albumin in PBST for 30 min at room temperature. The cells were hybridized with probe mixture (Biotin-labeled oligonucleotide probe against circRNA mSCAR) at 37℃ in dark overnight, and the cells were rinsed in SSC buffer. Subsequently, cells were incubated with anti-Tom 20 antibody (abcam, US) for 1 h at room temperature. Finally, nuclei were counterstained. The images were obtained by confocal microscope.

In vivo biodistribution of exosomes: The exosomes were labeled with DiR or DiI (Invitrogen, US) at the final concentration of 8 μM. After removing the free dyes, labeled exosomes were injected into mice. Different tissues were harvested 6 h after injection. For vivo fluorescence tracing, DiR labeled exosomes were visualized by imaging using the IVIS® Lumina II *in vivo* imaging system (PerkinElmer, Thermo Fisher, US). To view the exosome cellular uptake by macrophage in indicated tissue, tissue sections from mice treated with DiI labeled exosomes were prepared and additionally incubated with anti-F4/80 antibody (Abcam, UK), followed by staining of Hoechst. All images were obtained by Nikon A1 Spectral Confocal Microscope (Nikon, Japan).

Treatment regiments of septic mice: In therapeutic intervention using exosomes, $Exo^{Mito\text{-}circRNA\ mSCAR}$ was injected into the tail vein every 6 hours for a total of 4 times after the mice developed significant sepsis symptoms. As a control, same volume of Exo^{Ctrl} and $Exo^{circRNA\ mSCAR}$ was injected in the same manner. The survival rate was monitored every 4 h.

Serum biochemistry: Blood samples were collected and the concentration of ALT, CK, and Cr were measured by Chemray 800 at Wuhan Servicebio technology Co., Ltd.

Lung wet/dry weight: For assessment of CLP-induced lung edema, the lung tissues were harvested from mice and were immediately weighted to obtain the wet weight. Lungs was then dried in a thermostatic at 65℃ for 72 h and the dry weight was thus obtained. Then, lung wet/dry ratio was calculated.

Echocardiography: Cardiac function was assessed with echocardiography (Vevo 2100 high-resolution imaging system equipped with a 13-24 MHz transducer, FUJIFILM VisualSonics, Canada). The heart beats of mice were kept between 400-500 beats per minute during the examination. Parasternal long axis view, short axis view and four-chamber view were scanned for multiple cardiac functional parameters. Each parameter was repeatedly measured for 6 times. Investigators were blinded to the identify of animals.

HE staining: Tissues were harvested and fixed using 4% buffered formaldehyde and then embedded using paraffin and serially sectioned (4 μm) *in toto*. Each slide was stained with hematoxylin and eosin (H&E) for examination of morphological damage microscopically.

Statistical analysis: Data were analyzed with GraphPad Prism7 software. Student's t-test was used for two-group comparison. One-way ANOVA test with Tukey's post hoc test was used to compare data for more than two groups. Two-way ANOVA with Sidak's multiple comparisons test was used to regarding the time-dependent effects of sepsis on macrophage polarization. Data are expressed as mean ± S.E.M. Statistical significance was set at $P < 0.05$.

References

[1] D. Rittirsch, M. S. Huber-Lang, M. A. Flierl,P. A. Ward *Nature protocols* **2009**, *4*, 31.

[2] Y. G. Chen, M. V. Kim, X. Chen, P. J. Batista, S. Aoyama, J. E. Wilusz, A. Iwasaki,H. Y. Chang *Mol Cell* **2017**, *67*, 228.

[3] B. Li, L. Zhu, C. Lu, C. Wang, H. Wang, H. Jin, X. Ma, Z. Cheng, C. Yu, S. Wang, Q. Zuo, Y. Zhou, J. Wang, C. Yang, Y. Lv, L. Jiang,W. Qin *Nature communications* **2021**, *12*, 295.

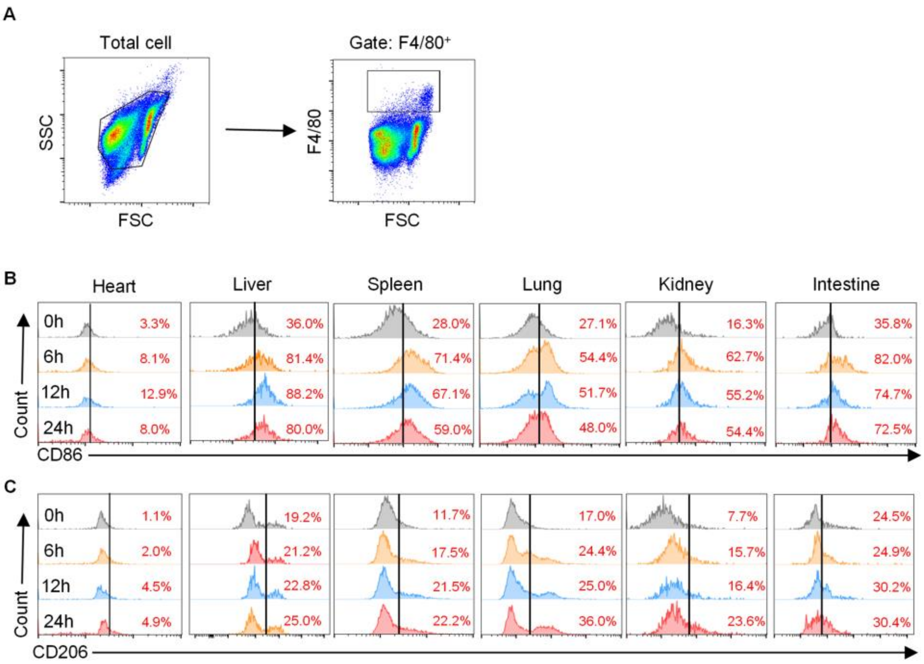

Figure S1. Imbalanced M1/M2 polarization in murine septic model. (A) Gating strategy for macrophage analysis. (B-C) Flow cytometry analysis of proinflammatory macrophages (F4/80$^+$ CD86$^+$) and anti-inflammatory macrophages (F4/80$^+$ CD206$^+$) population in indicated tissues of mice at 0 h, 6 h, 12 h, and 24 h after CLP. Representative images of 3 different experiments.

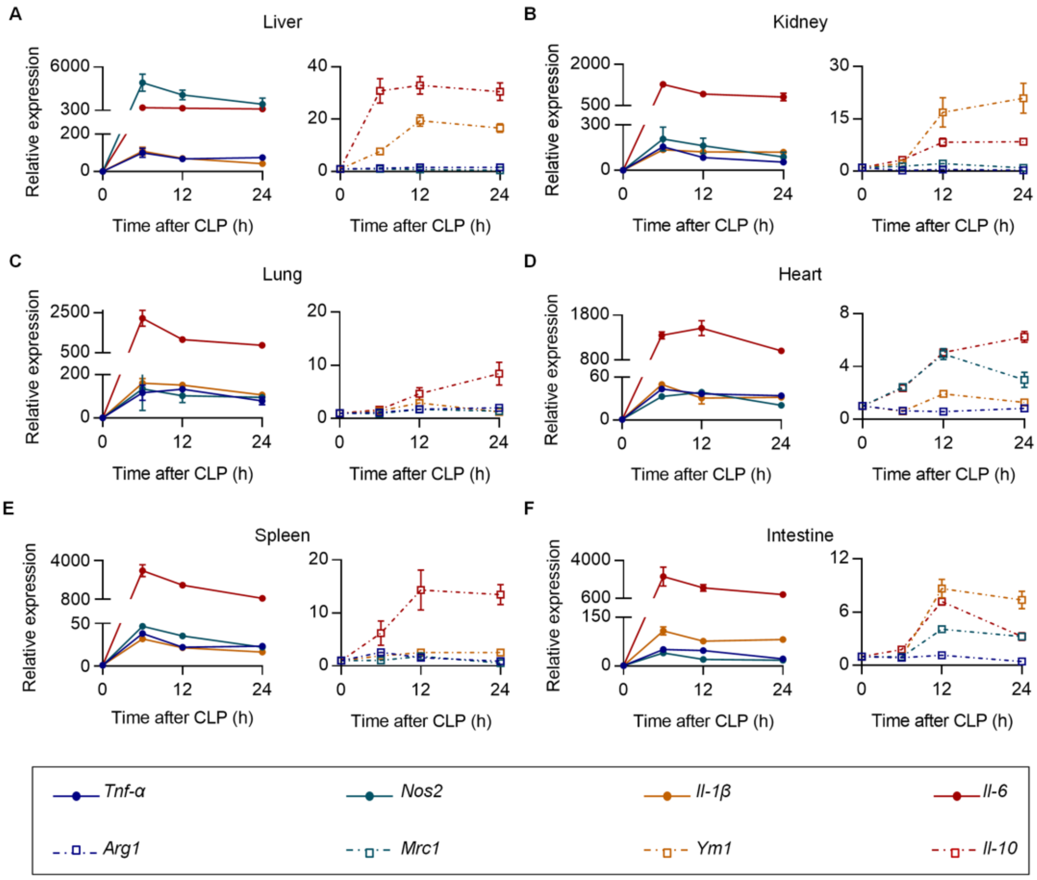

Figure S2. Excessive inflammation observed in septic mice. (A-F) Levels of proinflammatory cytokines (*Tnfα*, *Nos2*, *Il1β*, and *Il6*) and anti-inflammatory cytokines (*Arg1*, *Mrc1*, *Ym1*, and *Il10*) after

CLP treatment in interested tissues from septic mice. Data are present as means ± S.E.M of 3 biological replicates.

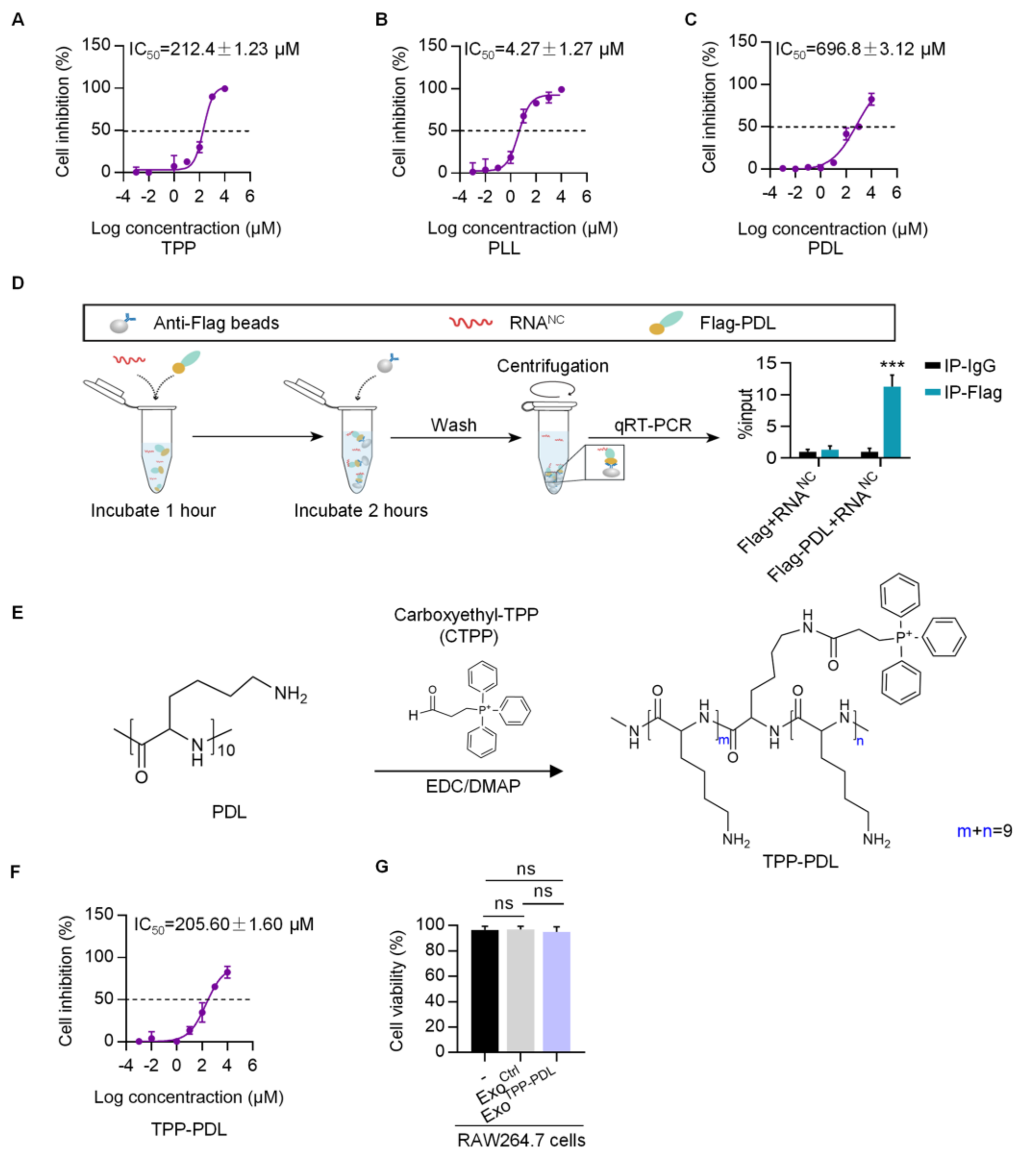

Figure S3. Synthesis of TPP-PDL and cytotoxicity analysis. (A) RAW 264.7 cells were treated with various concentrations of TPP. The cytotoxicity was detected by the CCK-8 assay. (B) CCK-8 assay of RAW 264.7 cells receiving indicated concentration of PLL. (C) CCK-8 assay of RAW 264.7 cells receiving indicated concentration of PDL treatment. (D) Schematic representation of the RNA-IP analysis to confirm the possible RNA/PDL interaction. Flag or Flag-PDL was incubated with RNA, followed by RNA-IP using IgG or anti-Flag antibody. RNA pulled down by Flag-PDL was analyzed by qRT-PCR. (E) Triphenylphosphonium (TPP) was chemically conjugated to PDL by EDC coupling. (F) CCK-8 assay of RAW 264.7 cells receiving indicated concentration of TPP-PDL. (G) RAW264.7 cells were treated with Exo^{Ctrl} and $Exo^{TPP\text{-}PDL}$ for 24 h. The cytotoxicity was detected by the CCK-8 assay. Data are expressed as means ± S.E.M. of three different experiments. ***p<0.001 by Student's t test or one-way ANOVA.

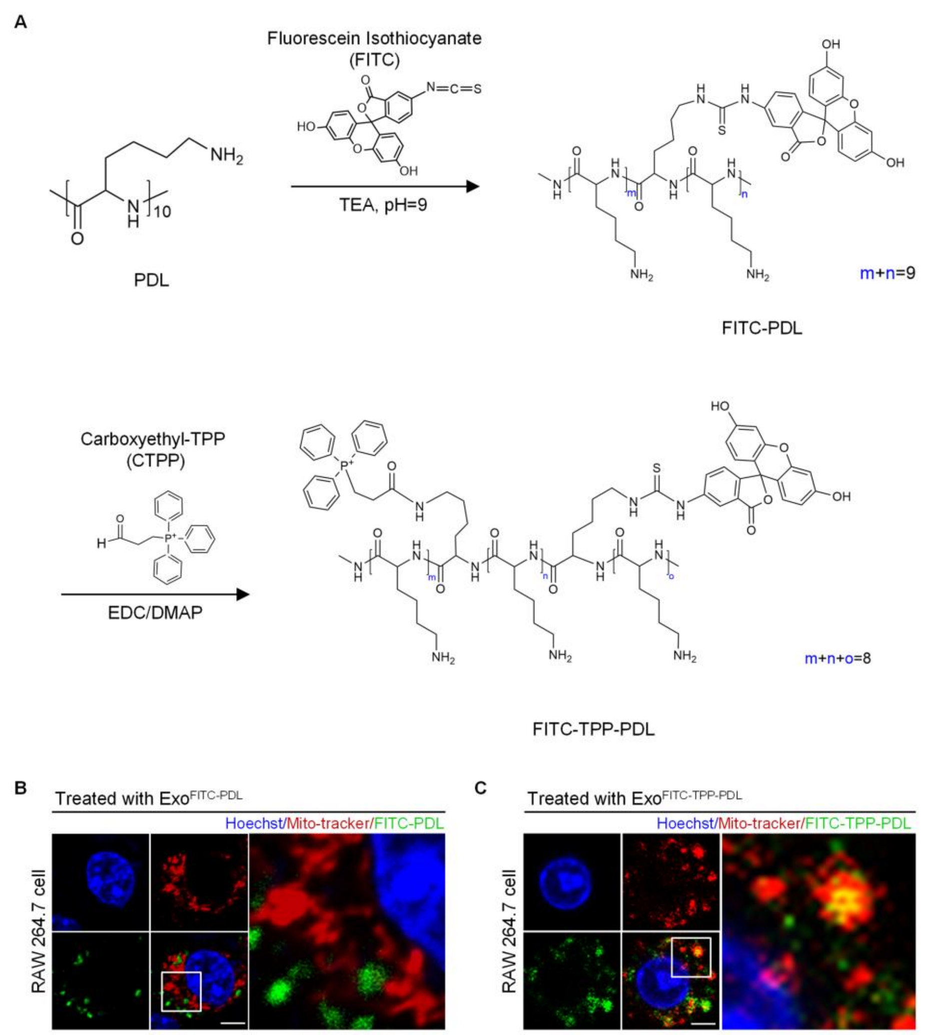

Figure S4. Exosome-based mitochondrial delivery of TPP-PDL in RAW 264.7 cells. (A) Schematic illustration of the synthesis of FITC conjugated TPP-PDL. (B-C) Representative confocal fluorescence microscopy images showing the distribution of PDL and TPP-PDL. RAW264.7 cells were treated with exosomes which containing FITC-labeled PDL or FITC-labeled TPP-PDL. The mitochondria were stained with MitoTracker (red), and nuclei were stained with Hoechst (blue). Scale bar, 5 μm.

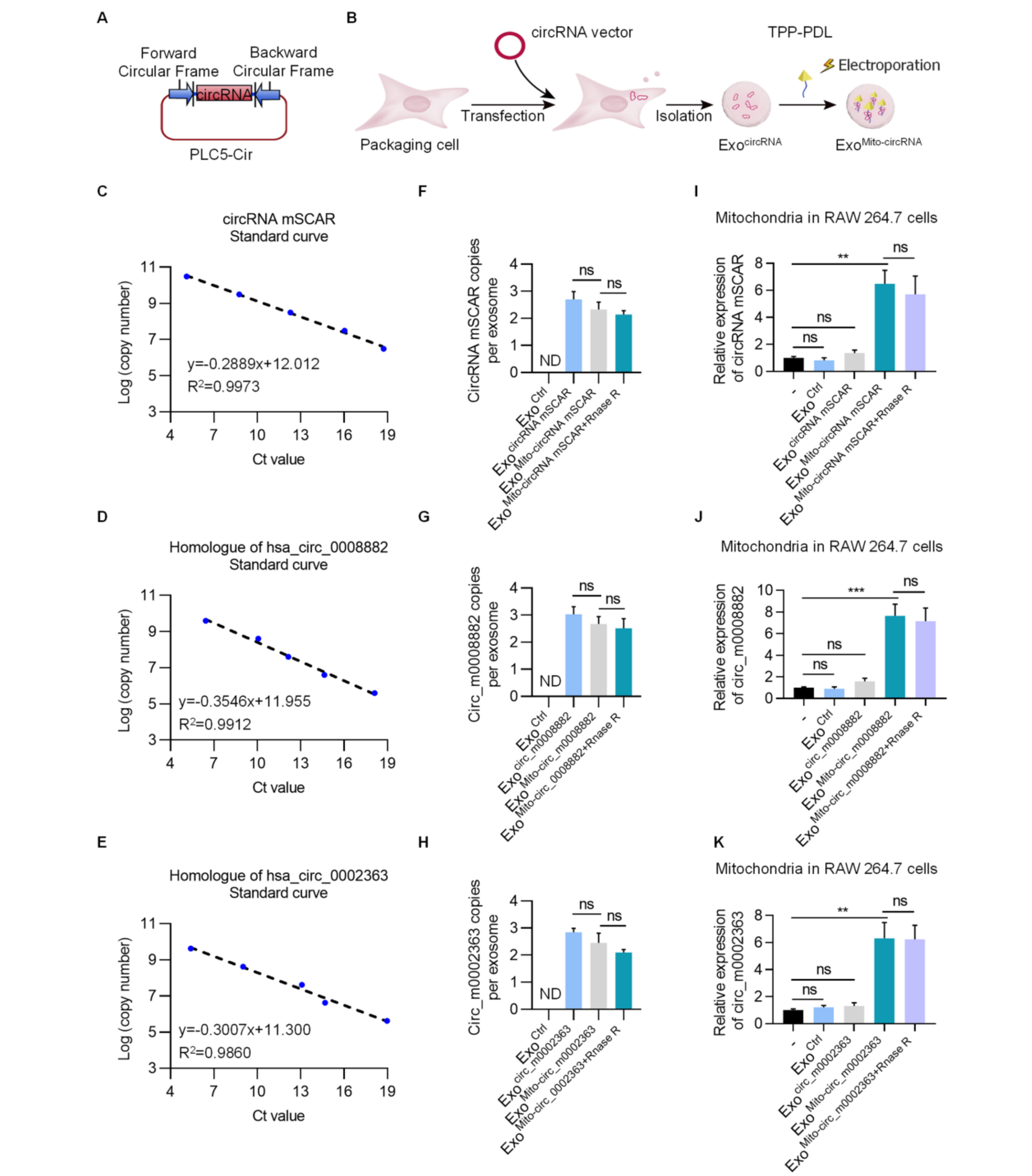

Figure S5. Exosome-based mitochondrial delivery of circRNA in to RAW264.7 cells. (A) Schematic illustration of the cloning of circRNA-expressing plasmids. (B) Schematic illustration of the procedure how circRNA was encapsulated and TPP-PDL was loaded by electroporation. (C-E) The standard curves of log circRNA copy numbers and the Ct values as determined by qRT-PCR. (F-H) Average circRNA copies per exosome as determined by absolute qPCR. (I-K) Levels of circRNA in mitochondria of macrophages treated with indicated, as detected by qRT-PCR. *Mtco₂* mRNA was served as the control. Data are expressed as means ± S.E.M. of three independent experiments. $**p<0.01$, $***p<0.001$ by one way ANOVA with Tukey's post hoc test.

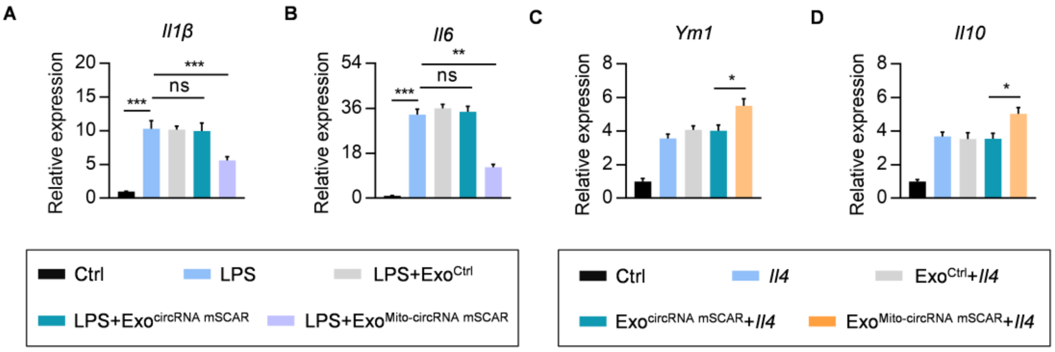

Figure S6. Mitochondrial specific delivery of circRNA mSCAR inhibits proinflammatory cytokine expression while increases anti-inflammatory cytokine expression. (A-D) qPCR analysis of proinflammatory cytokines (*Il1β* and *Il6*) levels and anti-inflammatory cytokines (*Ym1* and *Il10*) in RAW 264.7 cells treated as indicated. Data are expressed as means ± S.E.M. of three different experiments. **p*<0.05, ***p*<0.01, ****p*<0.001 by one-way ANOVA with Tukey's post hoc test.

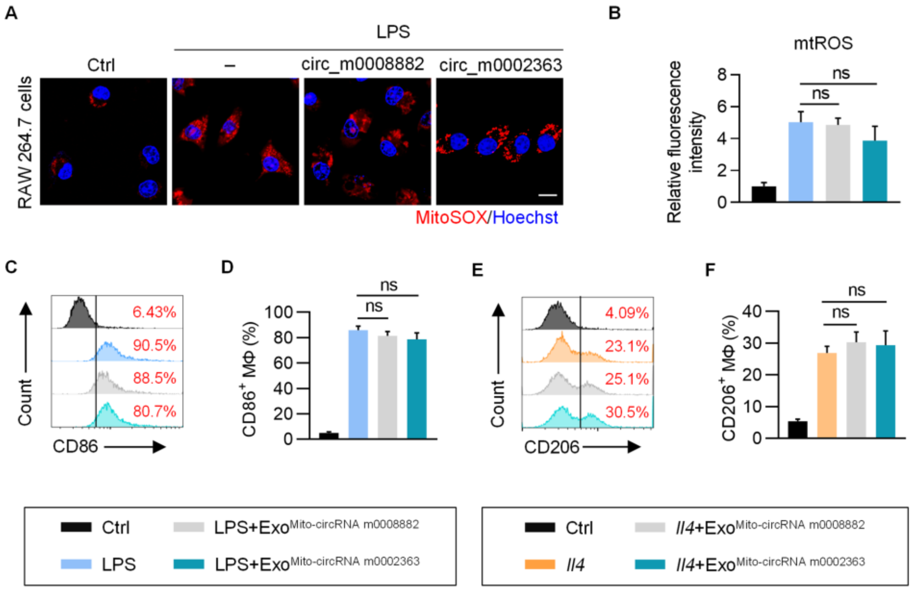

Figure S7. Circ_m0008882 and circ_m0002363 have no significant effects on mtROS and macrophage polarization. (A) Representative confocal images of MitoSOX in macrophages treated as indicated. Scale bar, 10 μm. (B) Quantitative fluorescence intensity was analyzed by Image J software. (C) Representative flow cytometry analysis of $CD86^+$ percentage in macrophages treated as indicated. (D) Quantification of $CD86^+$ percentage. (E) Representative flow cytometry analysis of $CD206^+$ percentage in macrophages treated as indicated. (F) Quantification of $CD206^+$ percentage. Data are expressed as means ± S.E.M. of 3 biological replicates. ns, no significant difference.

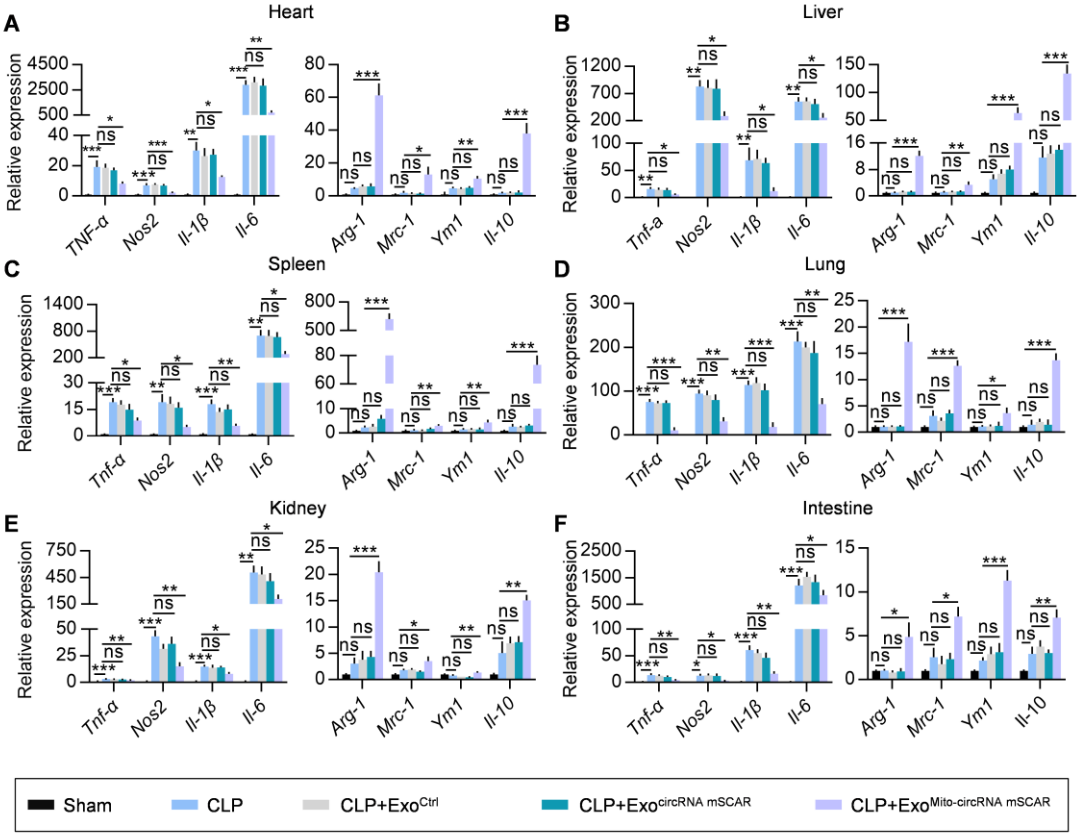

Figure. S8. Exosome-based delivery of circRNA mSCAR alleviates systemic inflammation in septic mice. (A-F) qPCR analysis of proinflammatory cytokines (*Tnfα*, *Nos2*, *Il1β*, and *Il6*) and anti-inflammatory cytokines (*Arg1*, *Mrc1*, *Ym1*, and *Il10*) in indicated organs from mice treated as indicated. Mice were subjected to CLP surgery, followed by injection of indicated exosomes. At the end of the experiments, mice were sacrificed and the tissues were harvested for qPCR analysis. Data are expressed as means ± S.E.M. of 4 biological replicates. **p*<0.05, ***p*<0.01, ****p*<0.001 by one-way ANOVA with Tukey's post hoc test.

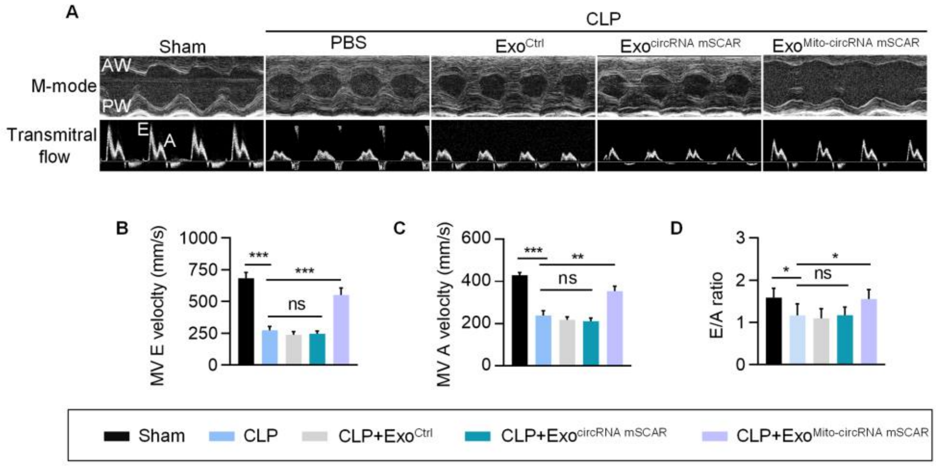

Figure. S9. Exosome-based delivery of circRNA mSCAR protects cardiac function in septic mice. (A) Representative images of M-mode echocardiographic and mitral inflow velocity in various groups treated as indicated. (B-D) MV E velocity (B), MV A velocity (C) and E/A ratio (D) were measured from mice in various groups. Data are expressed as means ± S.E.M. of 6 biological replicates. **p*<0.05, ***p*<0.01, ****p*<0.001 by one-way ANOVA with Tukey's post hoc test.

Table S1. The primer sequences

Name		Sequences
**qRT-PCR primers**		
Tnfα	Forward	5’-CAGGCGGTGCCTATGTCTCA-3’
	Reverse	5’-GCTCCTCCACTTGGTGGTTT-3’
Nos2	Forward	5’-GTTCTCAGCCCAACAATACAAGA-3’
	Reverse	5’-GTGGACGGGTCGATGTCAC-3’
Il1β	Forward	5’-CTCAACTGTGAAATGCCACC-3’
	Reverse	5’- GAGTGATACTGCCTGCCTGA-3’
Il6	Forward	5’-AGTTGCCTTCTTGGGACTGA-3’
	Reverse	5’-CAGAATTGCCATTGCACAAC-3’
Arg1	Forward	5’-CTCCAAGCCAAAGTCCTTAGAG-3’
	Reverse	5’-GGAGCTGTCATTAGGGACATCA-3’
Mrc1	Forward	5’-CTCTGTTCAGCTATTGGACGC-3’
	Reverse	5’-CGGAATTTCTGGGATTCAGCTTC-3’
Ym1	Forward	5’-CAGCTCCTCTCAAAAGGATGTG-3’
	Reverse	5’-CTTGGGCAAACTGCTATCAGTAT-3’
Il10	Forward	5’-TTAAGGGTTACTTGGGTTGC-3’
	Reverse	5’-GAGGGTCTTCAGCTTCTCAC-3’
β-actin	Forward	5’- GGCTGTATTCCCCTCCATCG-3’
	Reverse	5’-CCAGTTGGTAACAATGCCATGT-3’
U6	Forward	5’-CTCGCTTCGGCAGCACA-3’
	Reverse	Provided in the kit
$Mtco_2$	Forward	5'-AACAAAACTAACACATACAAGC-3'
	Reverse	5'-TAGGGGATGTGGCGTCTTGTA-3'
circRNA mSCAR	Forward	5’-GGGGATGTGGCGTCTTGTAG-3’
	Reverse	5’- CAACAACCCCGTATTAACCGT-3’
homologue of hsa_circ_0089761	Forward	5’-AATAGGGGATGTGGCGTCTT-3’
	Reverse	5’-TCCGCCCAATCACACAAATT-3’
homologue of hsa_circ_0008882	Forward	5’-CTCGCCCTCACAGGAATAC-3’
	Reverse	5’-CTAGGCATGATGATGTGAATG-3’
homologue of hsa_circ_0002363	Forward	5’-ACTGAAATAATTAGGGCTGTGGT-3’
	Reverse	5’-TTCCTAATCGCACTAGAACT-3’
RNA^{NC}	Forward	5’-CGTGACACGTTCGGAGAAT-3’
	Reverse	Provided in the kit
**Primers for plasmid construction**		
circRNA mSCAR	Forward	5’-CGGAATTCTAATACTTTCAGGGCCATAGAATAACCCTGGTC-3’
	Reverse	5’-CGGGATCCAGTTGTTCTTACTACCCATTCCAACTTGGTCTA-3’
homologue of hsa_circ_0008882	Forward	5’-GGCTCGAGAAAGTGCTGAGATTACAGGCG-3’
	Reverse	5’-GGGAATTCTGCTGGGATTACAGGTGTGAG-3’
homologue of hsa_circ_0002363	Forward	5’-GGCTCGAGAAAGTGCTGAGATTACAGGCG-3’
	Reverse	5’-GGGAATTCTGCTGGGATTACAGGTGTGAG-3’
**Primers for sequencing**		
circRNA mSCAR		5’-GGGCACCAATGATACTGAAGC-3’
homologue of hsa_circ_0089761		5’-AATAGGGGATGTGGCGTCTT-3’
homologue of hsa_circ_0008882		5’-CTCGCCCTCACAGGAATAC-3’
homologue of hsa_circ_0002363		5’-GGGCTGTGGTTTTTAAAAATCATGG-3’
**Oligos**		
RNA^{NC}		5’-UUCUCCGAACGUGUCACGUTT-3’

FAM-RNA^{NC}	5’-FAM-UUCUCCGAACGUGUCACGUTT-3’
Biotin-labeled oligo probe	5’-Biotin-GUUAUUCUAUGGCCUACCCAUUCCAACUUGG-3’
